# Recent Progress on Metal–Organic Framework and Its Derivatives as Novel Fire Retardants to Polymeric Materials

**DOI:** 10.1007/s40820-020-00497-z

**Published:** 2020-08-25

**Authors:** Jing Zhang, Zhi Li, Xiao-Lin Qi, De-Yi Wang

**Affiliations:** 1 IMDEA Materials Institute, C/Eric Kandel 2, 28906 Getafe, Madrid Spain; 2 Universidad Politécnica de Madrid, E.T.S. de Ingenieros de Caminos, 28040 Madrid, Spain; 3 China-Spain Collaborative Research Center for Advanced Materials, School of Materials Science and Engineering, Chongqing Jiaotong University, Chongqing, 400074 People’s Republic of China

**Keywords:** Metal–organic frameworks, Hybrids, Polymers, Composites, Fire retardancy

## Abstract

High flammability of polymers has become a major issue which has restricted its applications. Recently, highly crystalline materials and metal–organic frameworks (MOFs), which consisted of metal ions and organic linkers, have been intensively employed as novel fire retardants (FRs) for a variety of polymers (MOF/polymer). The MOFs possessed abundant transition metal species, fire-retardant elements and potential carbon source accompanied with the facile tuning of the structure and property, making MOF, its derivatives and MOF hybrids promising for fire retardancy research. The recent progress and strategies to prepare MOF-based FRs are emphasized and summarized. The fire retardancy mechanisms of MOF/polymer composites are explained, which may guide the future design for efficient MOF-based FRs. Finally, the challenges and prospects related to different MOF-based FRs are also discussed and aim to provide a fast and holistic overview, which is beneficial for researchers to quickly get up to speed with the latest development in this field.

## Highlights

- Recent advances of metal–organic frameworks (MOFs) in the fire-retardant polymeric materials are reviewed.
- State of the art to the novel strategies for functionalizing MOFs as fire retardants is critically and comprehensively discussed.

## Graphical abstract

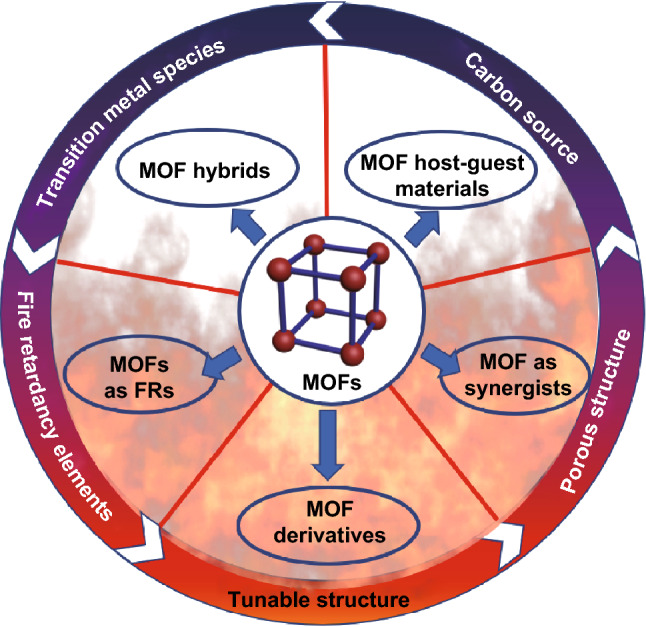

## Introduction

**Fig. 1:**
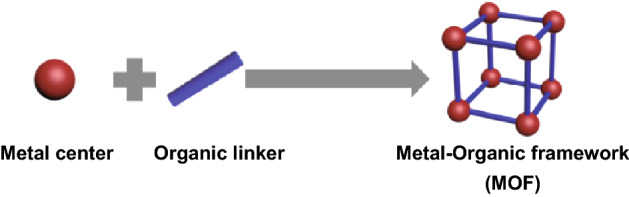
Schematic structure of the MOF

Metal–organic frameworks (MOFs) or porous coordination polymers (PCPs), which consist of metal centers and organic links (Fig. 1), have attracted great attention as a new high crystalline porous material. Based on different metal ions and organic linkers, the multi-component structure of MOFs allows for the opportunity of tuning the morphology and microstructures, which make MOFs ideal materials for targeted properties [1]. A series of key features such as high surface area, open metal site and high porosity make MOFs suitable in many advanced applications such as in batteries, sensors and catalysis [2–4]. In particular, MOFs have been proven to be a proper template for preparing functional micro-/nano-materials such as highly porous carbon materials [5]. The presence of the organic component of MOFs allowed the formation of carbon-based materials directly from the MOF such as both graphitic and amorphous carbon. Xu et al. [6] first synthesized nanoporous carbon with the use of MOF-5 as a sacrificial template with the presence of furfuryl alcohol. Nanoporous carbon was obtained with high BET surface by calcinating MOF at 1000 °C in Ar flow. Moreover, MOFs contains a variety of transition metals (e.g., Co, Ni, Cu, Fe) and fundamental elements (e.g., C, N, O) in the organic linkers for the catalytic applications [4]. By calcination of MOFs in different atmospheres such as air or N_2_ at an evaluated temperature, a series of MOF-derived hybrids such as metal oxide and metal/carbon were obtained by rational design of the MOF template and controlled thermolysis process [7]. The copper-based MOF (Cu-BTC)-derived Cu/Cu_2_O has been synthesized as catalysts toward carbon monoxide (CO) oxidation [8]. Co-based ZIF-67 was also reported to have high performance in CO oxidation at − 30 °C through facile pyrolysis in argon atmosphere [9]. Besides, apart from the conversion of poisonous CO to reduce the environmental impact, much effort has been devoted to making use of MOFs to adsorptively remove hazardous materials from fuel, water and air [10]. The metal ions, open metal sites, linkers and functionalization of MOFs showed different possible interactions such as unsaturated sites [11], *π*-complex formation [12] and hydrogen bonding [13].

Due to the high flammability of polymeric such as epoxy and PLA, the fire hazards including the release of heat, smoke, toxic gases and hypoxia (lack of oxygen) cause great loss to life and properties in the long history [14–16]. Previously widely applied halogen fire retardants are gradually banned in consideration of their threaten to health [17, 18]. Therefore, the emerging of metal-based inorganic FR [19, 20] and nanotechnology [21–23] have attracted a great attention. Recently, the increasing attention paid to the application of MOFs in fire retardancy research has been reflected by an increasing number of publications in various journals. The previously mentioned unique features of MOFs, such as adsorptive removal of hazardous materials, potential carbon source and effective catalytic performance, indicated the potential for using MOFs in the fire retardancy for the polymer composites. As a matter of fact, MOFs themselves or their functionalized derivatives may provide a platform for preparing a highly efficient FR compared with traditional FRs due (most likely) to the following several points: (1) abundant fire-retardant elements C, N, P in the organic linkers and transition metal species in the metal centers; (2) precursor of porous carbon in specified conditions; (3) possessing high surface area and microporous structure (< 2 nm [24]) to facilitate the adsorption for toxic gases; and (4) facile rational design of the microstructure, morphology and property through the choice of different metal centers and organic linkers and the possibility to further functionalize MOFs through coordination bond. Comparing with some inorganic FRs, MOFs as organic–inorganic hybrids possess the possibility to combine the properties of both inorganic and organic FRs. The presence of organic linker may enhance compatibility with polymer or relief the sensitivity of inorganic materials [25]. Therefore, suitable strategies for the design and synthesis of MOFs, MOF derivatives and their functionalized hybrids can be novel FRs for polymeric materials.

**Fig. 2:**
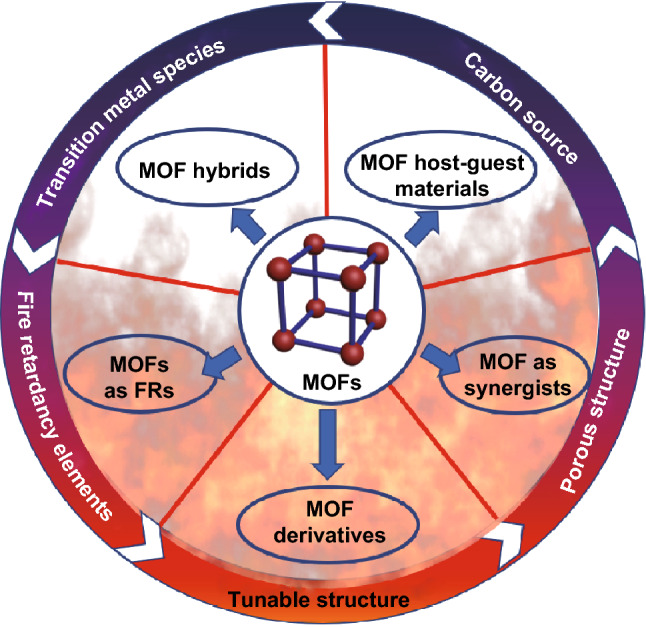
Schematic illustration of the potential advantages of MOF as FRs and a variety of methods for preparing MOF-based FRs

In this review, we summarize, for the first time, recent progress in fire-retardant polymer composites with the use of MOF-based FRs (MOF/polymer) as shown in Fig. 2. The strategies that utilize MOFs or their derivates are highlighted for a better understanding of the purpose of functionalization in microstructure component, thus beneficial for the further design. Then, the characterization, preparation methods, thermal and fire retardancy properties and mechanisms of MOF/polymer composites are also discussed.

## Thermal Properties of MOFs

Regarding the application of MOF as fire retardants for polymer composites, the thermal property of MOF is a crucial aspect to be considered either in the high-temperature polymer processing or in the possible degradation behavior in the fire. Therefore, studying the thermal property of MOF promoted the rational design MOF-based materials for target functions.

**Fig. 3:**
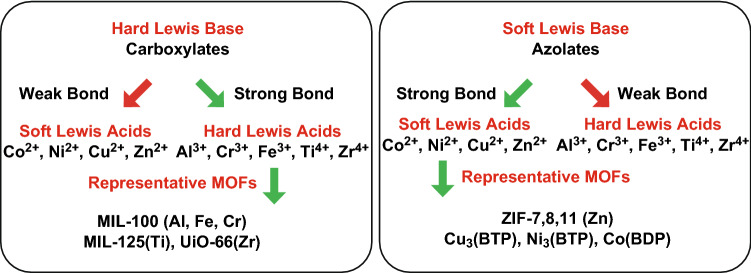
Stability of MOF guided by HSAB theory. Reproduced from Ref. [27] with permission from The Royal Society of Chemistry

Basically, the thermal stability of MOF can be predicted by the strength of the bonds of the formed structure [26]. The charge density including charges of metal source and ionic radius influenced the strength of bonds. Yuan et al. [27] proposed the strategy to construct stable MOFs based on Pearson’s hard/soft acid/base (HSAB) principle as shown in Fig. 3, in which high-valent metal ions with high charge densities can form stronger coordination bonds and thus a more stable framework. The frequently reported MOFs in fire retardancy research, such as ZIF-8 [28, 29], ZIF-67 [30] and UiO-66 [31–33], showed the decomposition temperature up to the range of 300 to 350 °C in both air and N_2_, respectively. Meanwhile, the processing temperature of polymers, especially thermoplastic polymers, can be up to 200 °C or even higher. Therefore, the choice of suitable thermal stable MOFs is the preliminary factor that has to be considered and it is feasible to meet this requirement for preparing polymer composites.

## Preparation of MOF/Polymer Composites

**Fig. 4:**
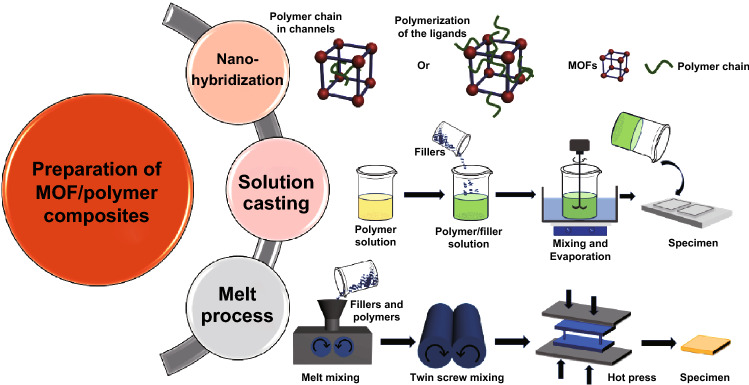
Schematic of the possible methods for preparing polymer@MOF composites

As shown in Fig. 4, there are various methods for preparing MOF/polymer composites from the reported literature. To achieve a well-dispersed state of fillers in the matrix, it is crucial to achieve the optimal property in both fire retardancy and mechanical property. Preparing MOF/polymer composites on different scales allows it to meet the requirements for diverse properties and applications. The details for preparing MOF/polymer composites are discussed in the following subsections.

### MOF and Polymer Nanohybridization

Encapsulating polymer chains into the regulated and adjustable nanochannels can achieve the precise control of the polymer chain distribution. It may further achieve the potential performance for the polymer composites compared with the polymer and MOF particles being randomly entangled in the bulk state [34]. Various synthetic methods have been applied such as the applied polymerization process within the channels of MOF [35], and polymerization of the ligands [36]. The materials prepared using this method have been applied in carbon dioxide (CO_2_) adsorption [37] and gas separation [38]. However, due to limitations in applying this method, applying it in large-scale industrial applications is difficult.

### Solution Casting Methods

Solution casting is a common fabrication method for preparing MOF/polymer composites. In general, MOF particles are first dispersed in the solvent through stir or sonication and then further mixed with the polymer solution for the final MOF/polymer solution. This is followed by heating at a certain temperature to fully remove the remaining solution. A “priming” technique is reported to obtain a well-dispersed solution, in which a small portion of polymer was mixed with MOF suspension with the addition of polymer to the required amount gradually [39]. The solution casting method is widely reported in the preparation of MOF-based mixed matrix membrane. Using the solution blending method to prepare polylactic acid (PLA) [40] and polystyrene (PS) [41], MOF composites were also reported. The better dispersion of the fillers in the polymer matrix can usually be obtained.

### Melt Compounding

The melt compounding method is much more suitable for commercial use due to it being environmentally friendly and versatile for larger scale processes compared with the two previously mentioned methods [42]. The melt blending method mentioned here includes the physical mixing of both thermoplastic and thermosetting polymer. By using this method, thermoplastic polymers such as PLA [43] or polypropylene (PP) were directly mixed with the melted polymer in a chamber followed by the physical mixing with the twin-screw extruder. The thermosetting polymers such as epoxy (EP) [44] or unsaturated polyesters (UP) [45] were melt-mixed with the fillers at high temperature followed by the curing process. The sheer force was beneficial in preventing the aggregation of the fillers within the matrix, which is widely reported in previous research for the preparation of the polymer composites in various applications.

## MOF Applied in Fire Retardancy Research

### MOF-Derived Materials as FRs

#### MOF-Derived LDH as FRs

**Fig. 5:**
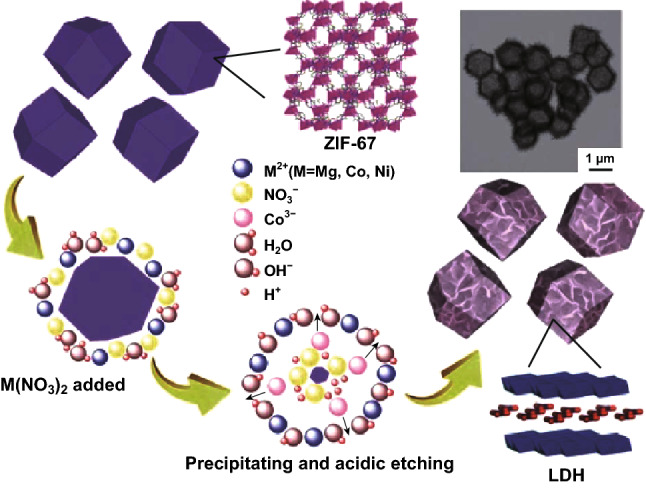
The formation illustration of LDH nanocages by simultaneous precipitation and acidic etching. Reproduced from Ref. [48] with permission from The Royal Society of Chemistry

Converting MOFs into well-known FRs such as layered double hydroxides (LDHs) is the initial process in exploring how to turn MOFs into FRs. Due to the specific feature of MOFs with reactive moieties, MOFs have demonstrated the suitable precursors and sacrificial template for preparing various hierarchical hollow materials such as porous carbon [46] and metal/carbon hybrids [47]. Among MOFs, two specific MOFs, ZIF-8, and ZIF-67, possessed the same topology structure, which are sensitive to the acidic and alkali conditions, thus providing the possibility of preparing a hollow structure in a facile way. Furthermore, cobalt ions with variable valences enabled the trivalent ions to become LDHs materials, which are a class of two-dimensional (2D) lamellar structure emerging as new inorganic FRs for polymers [19]. As shown in Fig. 5, Chen et al. [48] first reported the synthesis of LDH nanocage with the use of MOFs as templates which could be used as supercapacitors. The same strategy to prepare LDH from MOFs was later applied in fire retardancy research. Recent published papers from 2017 which focus on MOF-derived LDH and other materials as fire retardants applied in different polymers are summarized in Table 1.

**Table 1:** Summary of MOF-derived FRs on flame retardancy of polymer composites

Polymer	Derivation	Loading (wt%)	Type of FRs	Main fire retardancy results	References
EP (DDS)^a^	ZIF-67-derived NiCo-LDH	2.0	rGO@LDH	65.9% and 16.7% reduction in pHRR and TSP	[49]
EP (DDM)^b^	ZIF-8-derived pATH	20.0	DOPO-encapsulated pATH	65% reduction in pHRR	[55]
UPR	ZIF-67-derived NiCo-LDH	2.0	Graphene oxide@NiCo-LDH	35.5% reduction in pHHR	[50]
		2.0	Carbon nanotubes@NiCo-LDH	30.5% reduction in pHRR	
EP (DDM)	ZIF-67	4.0	Polyphosphazene@NiCo-LDH	30.9% reduction in pHRR	[52]
EP (DDS)	ZIF-67	2.5	MgAl-LDH@NiCo-LDH	UL-94 V0 ratio, 66.7% reduction in pHRR	[51]
EP (DDS)	ZIF-67-derived NiCo-LDH (NCH)	6.0	ZHS@ bimetallic (Ni-Co) hydroxides nanocage	UL-94 V0 rating, 69.1% reduction in pHRR	[53]

^a^DDS Diaminodiphenyl sulfone

^b^DDM Diaminodiphenyl methane

Pan et al. [49] proposed three-dimensional (3D) graphene/LDH hybrids (rGO@LDH) as high-performance FRs in epoxy nanocomposites. Acid-etched ZIF-67 stood and lied on graphene sheets, which benefited the restacking of graphene layers, thus enhancing the dispersion of fillers within the polymer matrix. With only 2 wt% of rGO@LDH, the peak heat release rate (pHRR) and total smoke production (TSP) of EP composites exhibited 65.9% and 16.7% reduction compared to that of pure EP. Moreover, the electrical resistivity of EP composites was also 81.4% higher than that of pure EP. This allowed the practical design of highly safe electrical insulating epoxy nanocomposites with fast heat dissipation and low fire hazards.

**Fig. 6:**
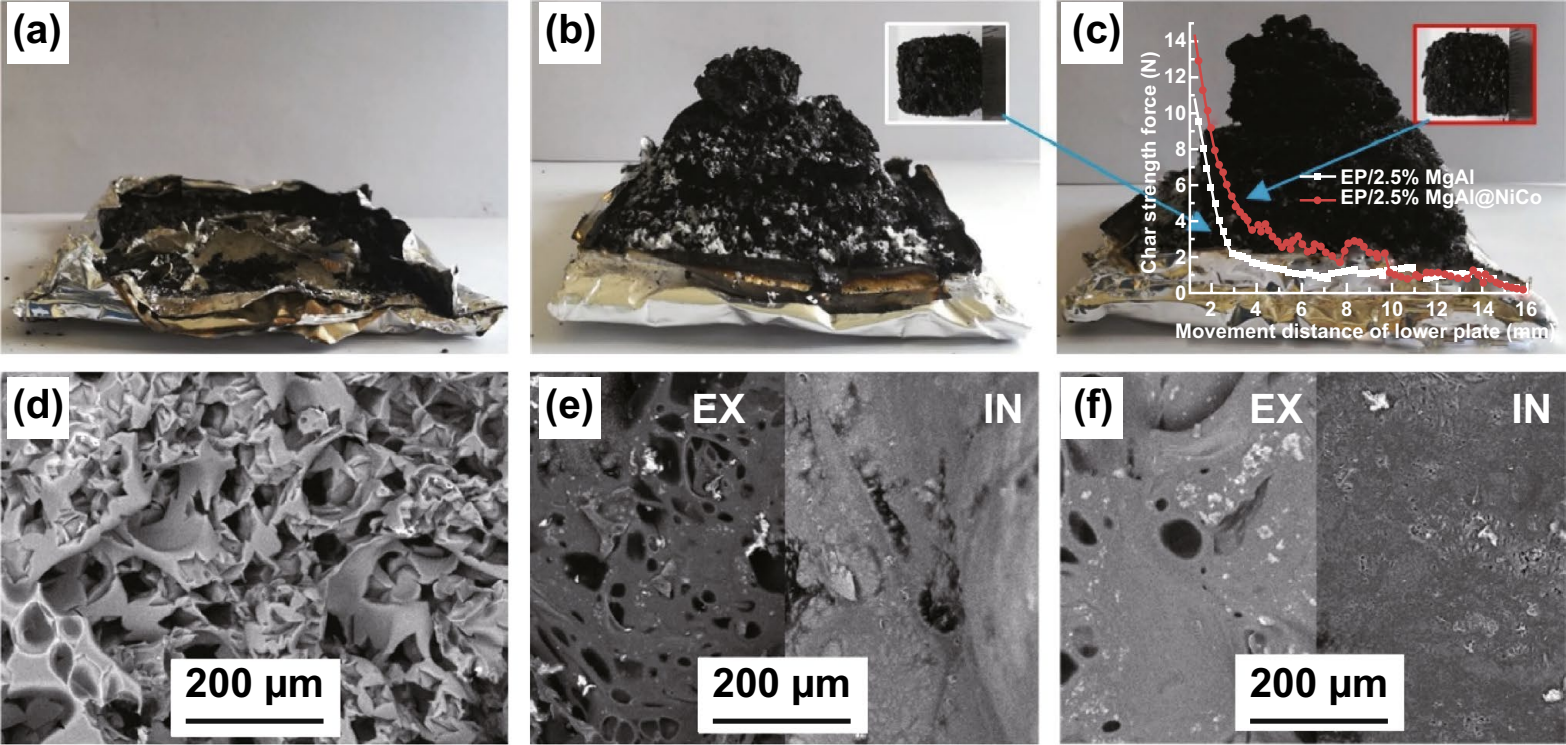
**a**–**c** Front view and **d**–**f** SEM images of char after the cone calorimeter test for EP, EP/2.5% MgAl, EP/2.5% MgAl@NiCo, respectively. Adapted from Ref. [51] with permission from Elsevier

The graphene oxide and carbon nanotubes surfaced anchored bimetallic-derived Co–Ni LDHs are reported to promote formation the compact char residue and reduce toxicity by catalytically reducing toxic CO yields (46.1% and 33.9% decreases in total CO yield) in unsaturated polyester resin (UPR) system [50]. Similar, the reinforced and intumescent char of EP composites was also observed when incorporating the MOF-derived dual MgAl-LDH@NiCo-LDH hybrids [51]. The embedded curves in Fig. 6c presented and measured the strength of the char which was improved. The microstructure with fewer pores in SEM images (Fig. 6d–f) further indicated this enhanced char residues. Other works in preparing different MOFs-derived LDH hybrids such as with polyphosphazene [52] and zinc hydroxystannate (ZHS) nanoparticles [53] exhibited great enhancement in reducing the fire hazards and improving fire safety for polymers.

#### MOF-Derived Aluminum Hydroxide as Fire Retardants

Magnesium hydroxide and aluminum hydroxide (ATH) are cost-effective inorganic fire retardants [54], which are usually added directly or together with other synergists into polymer. However, the great quantities required usually lead to a decline in the processability and mechanical properties of the polymer composites. The mesoporous aluminum hydroxide with tunable pore width by using MOFs as sacrificial templates due to their PH sensitivity features [55] was prepared. The high surface area of porous ATH (pATH) allowed a higher loading of phosphorus FRs (9,10-dihydro-9-oxa-10-phosphaphenanthrene-10-oxide, designated as DOPO). The EP/pATH-DOPO composites exhibited the great reduction of fire hazards, in which the pHRR and TSP decreased by 65% and 30%, respectively.

**Fig. 7:**
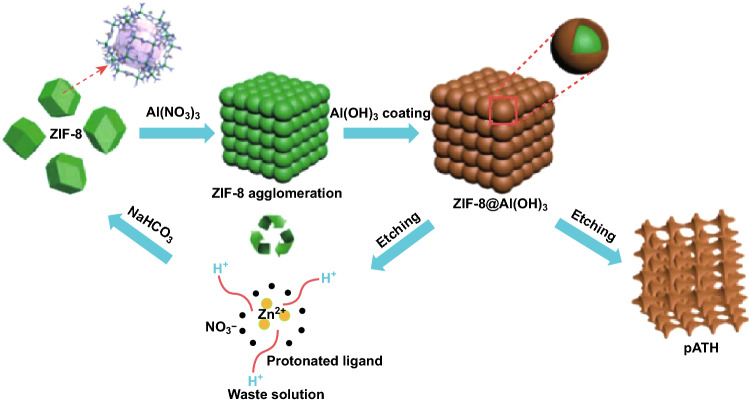
Schematic illustration of the synthetic procedure for pATH. Adapted from Ref. [55] with permission from the Royal Society of Chemistry

**Fig. 8:**
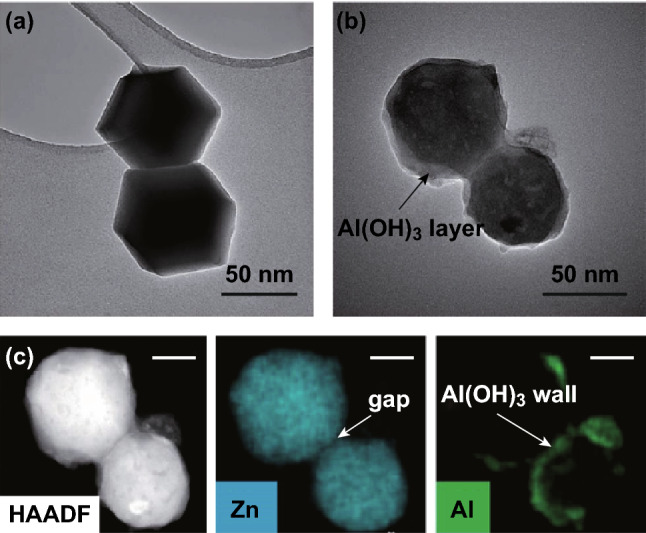
Investigation of the formation mechanism of pATH through TEM. **a** TEM image of the ZIF-8-2 h template; **b** TEM image of the intermediate gathered after a reaction time of 10 min; **c** HAADF image and elemental mapping (scale bar: 20 nm). Adapted from Ref. [55] with permission from the Royal Society of Chemistry

Moreover, the mechanical property of epoxy composites was studied by tensile tests and dynamic mechanical analysis (DMA), which exhibited the increased storage modulus due to the improved stiffness from the inorganic fillers. The slightly enhanced (diminished) tensile strength for EP composites with DOPO modified pATH (ATH) indicated better interaction of pATH with polymer matrix. Interestingly, a green and renewable cycle by the regeneration of the dissolved ZIF-8 in mild alkali conditions is available, which led to the deprotonation of the ligand and thus the preparation of pATH (Fig. 7). The corresponding formation mechanism of pATH was investigated by TEM and the elemental mapping result as shown in Fig. 8. With the disappearance of ZIF-8 in the interfacial region adjacent to the shell of ATH, newly generated ATH was supplied to fill the interface until termination of the hydrolysis reaction (amorphous replica method). This template-engaged nano-architecture method has the potential to advance the research in fire retardancy.

### MOF Directly as FRs

After exploring the conversion of template-engaged MOFs into a series of novel FRs with porous structure, MOFs itself equipped with various transition metal centers and catalytic carbonization features also showed potential as fillers for polymers. Hou et al. [41], for the first time, reported incorporation of iron-based and cobalt-based MOFs into polystyrene. The 14% and 28% reductions in pHRR were observed for PS/Fe-MOF and PS/Co-MOF composites compared with that of neat PS. They reported that the formation of porous metal oxides derived from MOFs acting as a thermal barrier is one of the factors in achieving the fire retardancy. Moreover, it showed the restraint for the release of toxic styrene oligomers. Various MOFs which consisted of different transition metal centers such as zinc-based ZIF-8 [56, 57], cobalt-based ZIF-67 [58], copper-based HKUST-1 [45], iron-based MIL101 (Fe) [41] and zirconium-based UiO-66 [33, 59] have been applied as FRs in different polymers which showed different levels of fire retardancy efficiency. The same MOF has led to significant differences in providing fire retardancy and suppressing smoke due to the different mechanisms based on the features of both polymer and MOF such as the chemical structure. For example, the PS composites with the presence of 5% zirconium-based MOF (UiO-66) exhibit 26.8% reduction in pHRR [59]. The addition of 4% Zr-MOF imparts polycarbonate (PC) 48% reduction in pHRR and UL-94 V0 rating. The unsaturated Zr metal site made MOF possess catalytic oxidation and char formation ability [33].

**Fig. 9:**
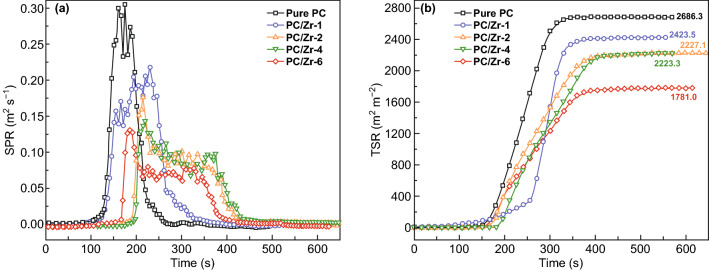
Smoke production rate (SPR) (**a**) and total smoke rate (TSR) (**b**) curves of PC composites. Adapted from Ref. [33] with permission from Elsevier

On the contrary, many MOFs showed similarity exhibiting capacity to lower the fire hazards such as suppress of smoke, toxic CO and PH_3_ which are reported in different polymer matrixes such as EP, PS and PA6 [33, 41, 60]. The 22.8%, 30%, and 28% reductions of TSP with the addition of only 2 wt% ZIF-8 [61], UiO66-NH_2_ [44] and ZIF-67 [62] for EP composites were reported. The total smoke rate (TSR) of PC composites is reported decrease by 17% with the presence of 2 wt% of UiO-66 (Fig. 9). The reduction in smoke demonstrated that a possible reason is due to catalytic capacity of MOF and the transition metal oxide derived from the MOF, which played a catalytic role such as cobalt oxide (Co_3_O_4_) [63–65]. The limitation of simply adding an MOF itself is usually not sufficient. Therefore, more research started to explore imparting polymer with highly efficient fire retardancy by further modifying MOFs using different methods.

### Hierarchical Functionalized MOF Hybrids as FRs

#### Phosphorus Modified MOF Hybrids

**Fig. 10:**
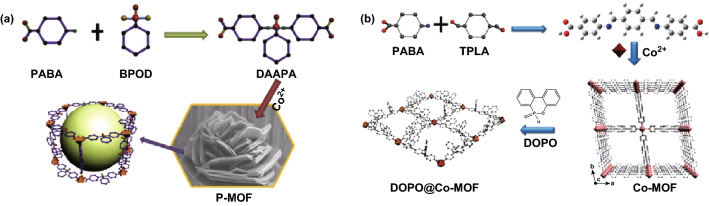
Two methods for preparing the phosphorus modified MOF as FRs. Reproduced from Ref. [25] with permission from Elsevier and Ref. [68] with permission from American Chemical Society

**Fig. 11:**
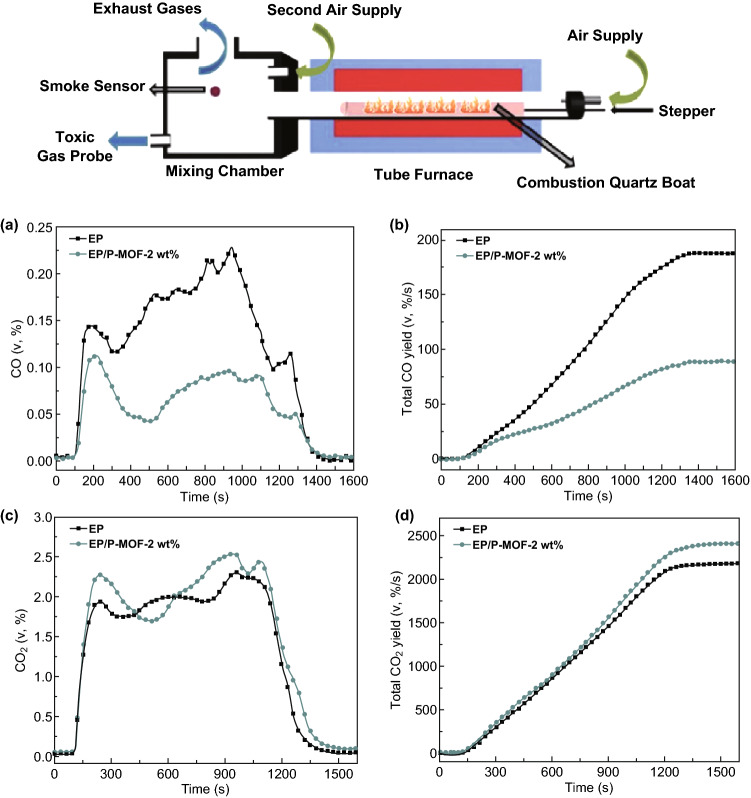
ISO 19700 steady state tube furnace apparatus (Top) and corresponded data of CO (**a**) and CO_2_ (**c**) and their total yield amount (**b**, **d**). Adapted from Ref. [25] with permission from Elsevier

To optimize the fire retardancy efficiency, the combination of phosphorus fire retardants with additional inorganic compounds is attracting increasing attention due to the phosphorous FRs influenced in both the condensed phase by enhancing the char and in the gas phase through flame inhibition. Among the inorganic materials, transition metal containing compounds are reported to functionalize with phosphorus FRs as a feasible way to prepare highly efficient FRs [66]. A ZIF-8 modified phosphorus containing layered α-zirconium phosphate (α-ZrP) was synthesized by electrostatic force [67]. Polyurethane (PU) composites with the addition of these fillers showed 45.6% and 40.6% reduction in pHRR and TSP, respectively. Moreover, Co-based MOFs abundant with transition metal species and phosphorus containing structure (denoted as P-MOF) were synthesized through a facile hydrothermal method and reported by Hou et al. [25] as shown in Fig. 10a. The layered MOF acted as a barrier and was able to provide a skeleton for char formation, thus suppressing the release of toxic gases such as CO for epoxy. The toxicity of gas was assessed by the effective apparatus, steady state tube furnace (SSTF) tests, based on standard ISO TS 19700 as shown in Fig. 11. The reduction yield of CO and increase in CO_2_ provide direct evidence of the excellent catalytic oxidation effect of P-MOF. The porous structure acted as a pathway for the absorption of degradation products. Hou et al. also reported another layered Co-based MOF (Co-MOF) with Schiff base as organic ligands which was designed and synthesized from para-aminobenzoic acid (PABA) and terephthalaldehyde (TPAL) as shown in Fig. 10b [68]. After modified Co-MOF with phosphorus fire retardants like DOPO, this novel MOF hybrid showed an enhancement in the fire retardancy and mechanical property for PLA.

**Fig. 12:**
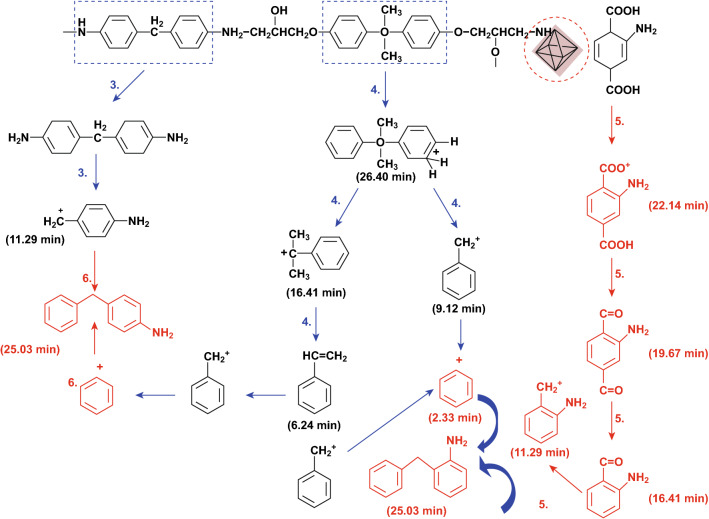
The new pyrolysis route of the EP composites with the addition of 5 wt% PA modified UiO66-NH_2_ sample. Reproduced from Ref. [44] with permission from American Chemical Society

The reduction of toxic CO with the combination of phosphorus modified MOF was also observed by Zhang et al. [44]. They reported the use of bio-mass phytic acid (PA) to functionalize zirconium MOF (PA-UiO66-NH_2_) as effective FRs to impart epoxy with enhanced fire retardancy. This resulted in a 65% reduction in the carbon monoxide production (COP) of the EP composites with the addition of 5 wt% of PA-UiO66-NH_2_. They reported that the existence of phosphate groups and amine groups on the FRs may react with the epoxy groups during the curing process which enhances the interfacial strength of fillers with polymer, and this is indicated by the decrease in the glass transition temperature (*T*_g_) and cross-linking density. They further proposed that the modified thermal decomposition of EP composites such as extensive random scission may enhance the char quality with higher polyaromatic structures, due to the interaction of the early decomposition products of EP and that of novel phosphorus functionalized MOF, which was evidenced by pyrolysis gas chromatography–mass spectrometry (Py-GC–MS). The possible modified pyrolysis route was provided as shown in Fig. 12. Indeed, thermal decomposition of phosphorus FRs and polymer as one of the factors affecting the fire retardancy performance of polymer composites and char formation is systematically studied [69]. This may also be applicable and should be considered when preparing phosphorus modified MOFs as highly efficient FRs.

#### Other Hierarchical MOF Hybrids

**Fig. 13:**
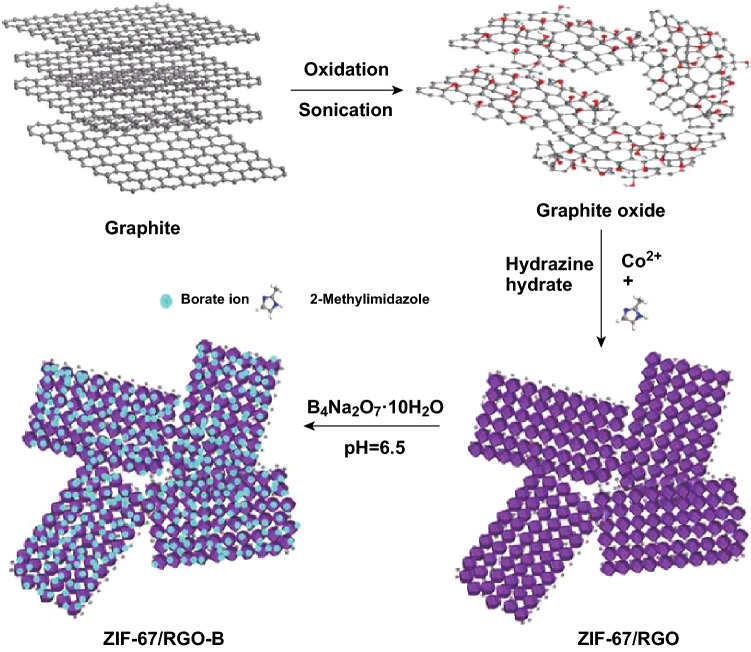
Scheme for preparing the borate ion modification of ZIF-67/RGO. Reproduced from Ref. [62] with permission from Elsevier

Graphene oxide (GO) as a two-dimensional material with a large surface area and rich functional groups is favored for the growth of MOFs due to the coordination bonds [61, 70]. MOFs and graphene oxide hybrids have been prepared and used in many different applications [71, 72]. Recently, they have also been attractive as FRs for EP and PLA, respectively. The results revealed that a 65% reduction of pHRR for epoxy was achieved, while the LOI value of the PLA/MOF@GO composites increased to 24% from 21% [73]. Moreover, the growth of bimetallic MOF on GO layer can be adjusted through controlling the ratio of Zn and Co sources [74]. The homogenous covered MOF@GO hybrids can further improve the fire retardancy of EP composites leading to an LOI value up to 29% and a 37% reduction in the production of carbon monoxide (COP). Adding borate ions adsorbed MOF-functionalized GO (ZIF67/RGO-B, Fig. 13) into epoxy can further enhance its fire safety, which exhibited a 65% reduction in pHRR and 66% reduction in smoke density [62]. They proposed that the fire retardancy mechanism was attributed to both the barrier effect of GO, and the formation of dense char, with the latter being due to Co_3_O_4_ derived from MOF. The inert gases such as N_2_ and NH_3_ also helped to dilute oxygen and flammable gases. Comparing fire retardancy results with the other GO hybrid FRs in EP, such as the pHRR reduction of GO hybrids with MCM-41 (40%) [75] or LDH (25%) [76], MOF@GO hybrids exhibit similar and even more attractive performance.

**Fig. 14:**
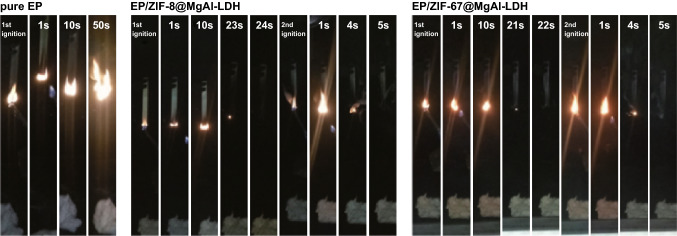
Digital photographs of EP, EP/ZIF-8@MgAl-LDH and EP/ZIF-67@MgAl-LDH during the UL94 vertical burning test process. Reproduced from Ref. [77] with permission from Elsevier

Hybridization of MOF with other fire retardants based on the different interactions between MOF and FRs was also reported to be an effective method. Through the electrostatic interactions, MOF and MgAl-LDH hybrids, ZIF-8@MgAl-LDH and ZIF-67@MgAl-LDH, were prepared to impart epoxy composites with enhanced fire retardancy, which were measured by the cone calorimeter test (CCT), UL-94 and LOI [77]. The reduction in the burning time in the UL-94 test was observed, which reached UL-94 V1 rating as shown in Fig. 14. Guo et al. [78] prepared silicon dioxide (SiO_2_, core) and zirconium-based MOF (UiO-66, shell) structure, where the two layers were connected through the covalent bonds (SiO_2_@UiO-66). Adjusting the ratio of the two materials can control the morphology of the hybrids. The 31% and 16% reductions in pHRR and TSP for EP/SiO_2_@UiO-66 composites indicated the improved performance in suppressing heat and smoke compared with any single component.

**Fig. 15:**
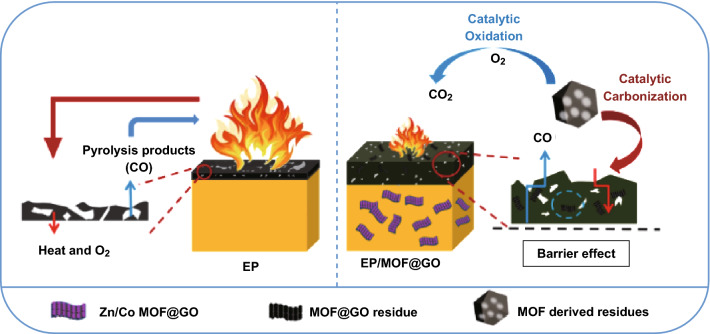
Proposed fire retardancy mechanism of EP/MOF@GO. Reproduced from Ref. [74] with permission from Elsevier

It is noticeable that MOF or MOF hybrid fire retardants exhibited more significant results in CCT than the small external heat flux fire tests (LOI and UL-94). This is also observed in other nano-FRs system due to barrier vanished for the nanocomposites system in small external heat flux leading to not significant improvement in the UL-94 [79, 80]. In the above studies, the MOF mainly influences the fire retardancy in two aspects as shown in Fig. 15 [74]: (1) catalytically promoting the formation of dense and thermal stable char, which acts as a barrier to protect the polymer from the heat and oxygen; and (2) suppressing the production of smoke and toxic CO through its catalytic oxidation behavior. The applications of MOF hybrids as FRs for polymer composites are summarized in Table 2.

**Table 2:** Summary of MOF hybrids as FRs on fire retardancy of polymer composites

Methods	Polymer	Loading (wt%)	Type of FRs	Main fire retardancy results	References
Phosphorus modified MOF	EP (DDM)	2	P containing MOF structure	28% and 18% reduction in pHRR and THR	[25]
	PLA	2	Co-MOF@DOPO	27% and 56% reduction in pHRR and TSP	[68]
	EP (DDM)	5	UiO66-NH_2_@phytic acid	41% and 42% reduction in pHRR and TSP	[44]
Hybridization of MOF	EP (MOCA)^a^	2	ZIF-8@MgAl-LDH	50.9% reduction in pHRR UL-94 V1 rating	[77]
			ZIF-67@MgAl-LDH	62.3% reduction in pHRR UL-94 V1 rating	
	PS	1	Ni-MOF@GO	33% and 21% reduction in pHRR and TSP	[70]
	PUE	2	ZIF-8@α-ZrP	69.6% and 40.5% reduction in pHRR and TSP	[67]
	EP (MOCA)	2	ZIF-8@rGO	65% and 37% reduction in pHRR and TSP, UL-94 V1 rating	[61]
	PLA	0.5	ZIF-8@GO	LOI value 24.5% (That of pure PLA 21%)	[73]
	EP (DDM)	3	UiO66-NH_2_@epoxy terminated SiO_2_	31% and 16% reduction in pHRR and TSP	[78]
	EP (MOCA)	2	ZIF67/RGO-B	65.1% reduction in pHRR, UL-94 V0 rating	[62]

^a^MOCA: 3,3′-Dichloro-4,4′-diaminodiphenyl methane

### MOFs or MOF Derivatives as Synergists

**Fig. 16:**
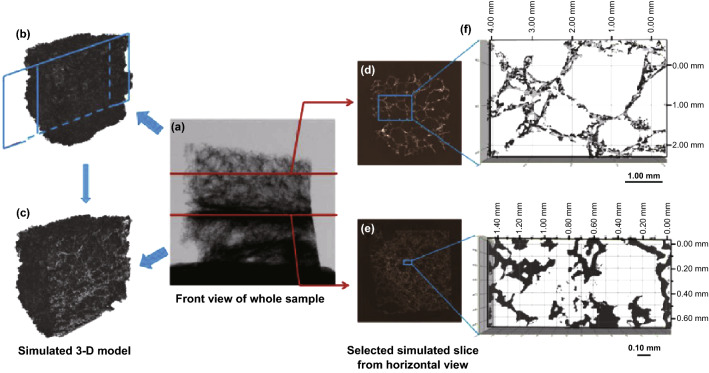
The images and simulated model from X-ray tomography: **a** front view of whole sample; **b**–**c** simulated 3D model of the whole sample and inner structure; **d**–**g** simulated slice at selected position from the horizontal view. Reproduced from Ref. [81] with permission from Elsevier

**Fig. 17:**
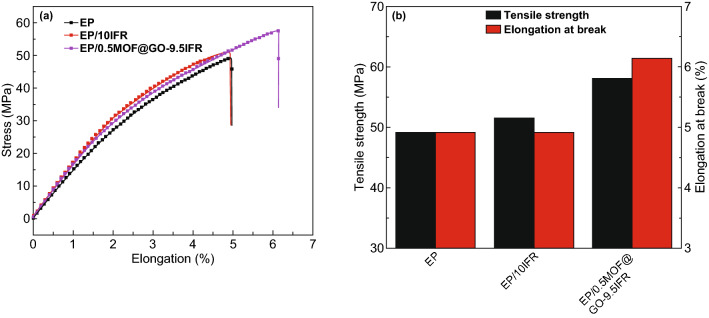
The mechanical property of EP composites by tensile test. Reproduced from Ref. [81] with permission from Elsevier

The optimization of the synergists based on traditional intumescent fire retardants (IFRs) system has attracted attention in the past two decades as a way to achieve a superior fire retardancy property. MOFs were applied as novel synergists to control the fire retardancy, mechanical property and carbonization process. Zhang et al. [81] first applied only 0.5% of bimetallic MOF with zinc and cobalt transition metal centers in the intumescent epoxy with significant improvement. Further, using bimetallic MOF and graphene oxide hybrids (MOF@GO) as synergists exhibited a 41% decrease in pHRR and 30% decrease in TSP. The systematic study for the carbonaceous char residue by XRD, in situ char morphology observation and X-ray tomography found a novel carbonization forming an alternative loose and accumulated structure as shown in Fig. 16. The study showed that the reinforced char structure with a good insulation property is crucial for achieving a good fire retardancy property. The mechanical property of EP composites did not deteriorate according to the tensile test result in Fig. 17, which is probably due to the high specific surface area and inorganic–organic hybrid feature of MOF, which can enhance the interfacial interaction [81].

**Fig. 18:**
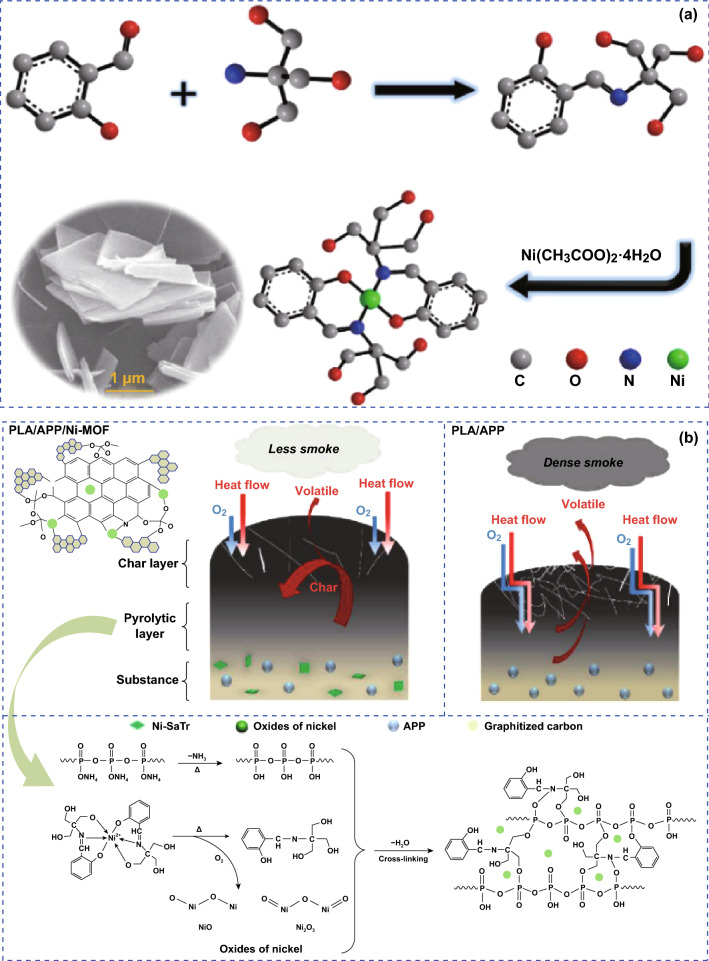
**a** Graphic illustration of preparation process of Ni-MOF. **b** Illustration of flame-retardant and smoke suppression mechanism. Adapted from Ref. [43] with permission from Elsevier

Moreover, the synergistic effect of MOFs with the combination ammonium polyphosphate (APP) on the polymer PLA was also studied. Firstly, the modified MOF (α-Phenyl-*N*-(2-propyl-2-hydroxymethyl-1, 3-dihydroxy)-imine-nickel (II), labeled as Ni-MOF) was prepared, which contained polyhydroxy groups. With the addition of total loading at 5 wt% (1.7% Ni-MOF and 3.3% APP), the fire safety PLA composites were achieved, which achieved UL-94 V0 rating [43]. They also reported that the dehydration and crosslink of polyhydric resulting from the decomposition of Ni-MOF are beneficial to the formation of the thermal stable char residue with the presence of more P and N elements, which act as a physical barrier in the condensed phase. The synthesis process of the Ni-MOF and its mechanism in the PLA composites are shown in Fig. 18. Other works related to the synthesis of nickel-based MOF-derived nickel phosphate as synergists were also reported to impart intumescent flame-retardant wood fiber/PLA composites toward fire safety [82]. The current reported work relating to the application of MOF-derived synergists is summarized in Table 3, including the composition as well as the main results in fire retardancy.

**Table 3:** Summary of MOF-based synergists on flame retardancy of polymer composites

Polymer	Type and loading of FRs and synergists	Main fire retardancy results compared with pure polymer	References
EP (DDS)	IFR (9.5 wt%) + Zn/Co MOF (0.5 wt%)	UL-94 V1 rating	[81]
	IFR (9.5 wt%) + Zn/Co MOF@GO (0.5 wt%)	UL-94 V1 rating pHRR and TSP reduction = 42% and 50%, respectively	
PLA	APP (5 wt%) + Ni-MOF (1 wt%)	UL-94 V2 rating	[43]
	APP (3.3 wt%) + Ni-MOF (1.7 wt%)	UL-94 V0 rating pHRR and TSP reduction = 27% and 50%, respectively	
PLA	Wood (25 wt%) +APP (5 wt%) + Ni-PO (5wt %)	UL94 V2 rating, LOI = 26.3%	[82]

**Fig. 19:**
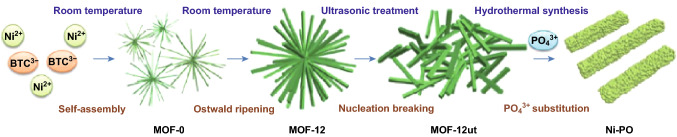
Schematic illustration of the formation from Ni-MOF to Ni-PO. Reproduced from Ref. [82] with permission from American Chemical Society

Aside from directly applying MOF as synergists with the incorporation of IFR, the MOF-derived nickel phosphate was also reported to have an effect in reducing the smoke in the intumescent fire-retardant wood/PLA system. The facile hydrothermal synthesis method shown in Fig. 19 prepared microsized rod-like nickel phosphate and also exhibited an enhancement in the mechanical property of PLA composites, for example, Young’s modulus.

### MOF Host–Guest Interaction

**Fig. 20:**
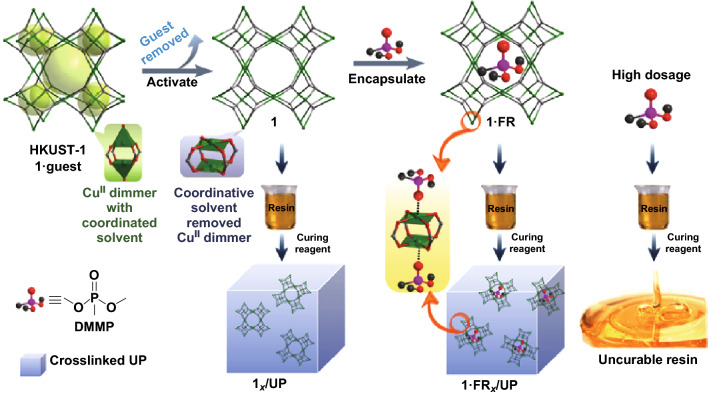
Scheme for encapsulating FR into MOF and UP composite preparation [26]. Reproduced from Ref. [45] with permission from American Chemical Society, https://pubs.acs.org/doi/10.1021/acsami.9b02357. Further permission related to the material excerpted should be directed to the ACS

Taking advantage of the highly porous inner structure of MOFs and incorporating FRs molecules into MOF host materials are feasible ways to avoid the release of volatile organic compounds (VOCs). Qi et al. [45] reported the infusion of phosphorus FRs-dimethyl methylphosphonate (DMMP) into porous copper-based MOFs HKUST-1 and then incorporated into unsaturated polyester (UP). The single-crystal X-ray diffraction, thermogravimetric analysis (TGA) and computational model proved that around 41% DMMP were encapsulated inside the host MOF particles due to the presence of open metal sites (OMSs) in HKUST-1 (Fig. 20). This hierarchical strategy simultaneously improved the fire safety of UP and its mechanical property. Similar strategy was also reported by encapsulating P and N containing ionic liquid (IL) into MOF (NH2-MIL-101(Al)) as novel FR for EP [83], which solved the poor dispersion and neutralizing the effect of directly adding IL into polymers. This IL-modified MOF combined the advantages of both materials and exhibited the 51% and 37.8% reduction in pHRR and SPR, respectively.

**Fig. 21:**
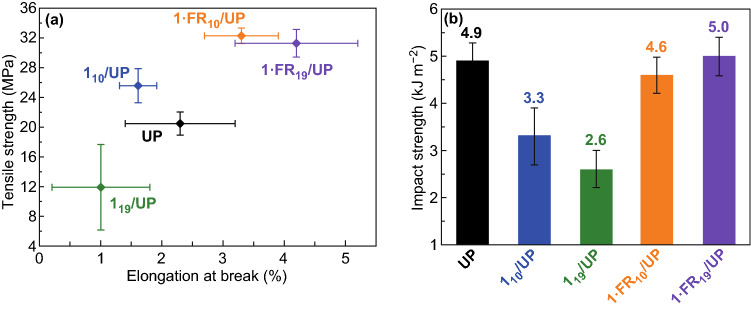
**a** Tensile properties and **b** impact strengths of UP and composites. Reproduced from Ref. [45] with permission from American Chemical Society, https://pubs.acs.org/doi/10.1021/acsami.9b02357. Further permission related to the material excerpted should be directed to the ACS

The addition of FRs at a high dose usually leads to the deterioration of the mechanical property. However, by encapsulating the DMMP into MOF, the similar impact strength and enhanced tensile strength, as shown in Fig. 21, indicated that the host–guest interaction eliminated the plasticization effect of DMMP. In summary, this hierarchical functionalization strategy by using MOF as host materials allowed the original fire retardancy mechanism of the polymer composites to strengthen and led to the effective improvement of polymer composites simultaneously in both the fire retardancy and mechanical property.

## Challenges and Current Limitations

Although many efforts have been tried as summarized to applying MOF as FR or FR component for polymers, there are a few drawbacks regarding the fire retardancy application that have to be faced critically in consideration of the aspects in practical concern, efficient design or future guideline. The detailed concerns were listed as follows:

1. The limited fire retardancy efficiency of MOF itself demonstrated that though it possesses the potential features, MOF directly as FR does not quite meet the high standard in the fire retardancy field.
2. Despite the large population of MOFs that have been synthesized and reported up to now relating to target functions, only a small number of MOFs have been applied as fire retardants. This is due to the fact that only MOF with certain features (e.g., specific metal centers, organic linkers, pore dimension, chemical and thermal stability of the framework) is suitable to be used in the fire retardancy field.
3. Due to the chemical structure of different polymers, each polymer exhibited a significant difference in the thermal degradation process. Therefore, the same MOF-based FRs exhibited a significant difference in the mechanism and performance for different polymer materials. Generally commenting efficiency of MOF FRs is difficult based on the current stage of studies have been explored.
4. The productivity and the cost-effective factors of MOF limited MOF as commercial materials in many practical applications.

## Conclusion and Prospects

In this review, the current state of the art in fire-retardant MOF/polymer composites is summarized. Although it is still in its early stages, MOFs have been proven to be valid components in achieving enhanced fire retardancy and suppression of smoke for polymers through MOF, its derivatives or MOF hybrids. This paper has reviewed the recent findings in relevant publications in this specific topic which are still quite fresh but provide an extremely attractive research direction. It is hoped that this can serve as a reference to guide researchers in determining the suitable methods for their work in this area. Various MOFs and new synthesized approaches have been developed to MOF-based hierarchical structure with the combination of effective fire retardancy component. In principle, MOFs are superior to other inorganic FRs since MOFs simultaneously possess a few advantages toward efficient FRs: potential carbon source, various pore geometries and facile functionalization. It is generally reported that the fire retardancy mechanism can be attributed to the following several aspects: (1) char formation; (2) catalytic oxidation; and (3) adsorption attributed to porous structure.

So far, various polymers such as PLA, epoxy, PS, PC and PU have been explored as fire-retardant MOF/polymer composites and they have shown an enhanced fire-retardant property. Nevertheless, MOF-based FRs are currently still in their infancy and are far from satisfactory. More future work may take effort in the serval aspects. Firstly, rationally choosing suitable MOF and applied functionalization strategy in different polymer matrix is a fundamental aspect to be considered. Secondly, a deep understanding of the structure–property relationship in MOF/polymer composites is highly essential. Roles of the porous structures, transition metal species, organic ligand species of MOF to fire retardancy of polymers should be further clarified in the future. For example, studies on a matched thermal degradation behavior between MOF and polymer matrix or adsorption ability for hazard gases are a promising way to guide the future design of MOF-based FRs. On top of the improved flame retardancy, since MOFs are versatile via tuning basic components (e.g., metal species, organic ligand species, porous structures, substituent group and nano-hybridization), they have great potential to act as multifunctional additives for polymers to achieve some advanced properties. As a result, we do believe that these are ambitious yet achievable prospects for MOF-based fire-retardant polymer composites.

## References

[1] Cao X, Tan C, Sindoro M, Zhang H (2017). Hybrid micro-/nano-structures derived from metal–organic frameworks: preparation and applications in energy storage and conversion. Chem. Soc. Rev..

[2] Zhao R, Liang Z, Zou R, Xu Q (2018). Metal–organic frameworks for batteries. Joule.

[3] Kreno LE, Leong K, Farha OK, Allendorf M, Van Duyne RP, Hupp JT (2012). Metal–organic framework materials as chemical sensors. Chem. Rev..

[4] Chen Y-Z, Zhang R, Jiao L, Jiang H-L (2018). Metal–organic framework-derived porous materials for catalysis. Coord. Chem. Rev..

[5] Chaikittisilp W, Ariga K, Yamauchi Y (2013). A new family of carbon materials: synthesis of MOF-derived nanoporous carbons and their promising applications. J. Mater. Chem. A.

[6] Liu B, Shioyama H, Akita T, Xu Q (2008). Metal–organic framework as a template for porous carbon synthesis. J. Am. Chem. Soc..

[7] Mai HD, Rafiq K, Yoo H (2017). Nano metal–organic framework-derived inorganic hybrid nanomaterials: synthetic strategies and applications. Chem. Eur. J..

[8] Zhang R, Hu L, Bao S, Li R, Gao L, Li R, Chen Q (2016). Surface polarization enhancement: high catalytic performance of Cu/CuOx/C nanocomposites derived from Cu-BTC for CO oxidation. J. Mater. Chem. A.

[9] Wang X, Zhong W, Li Y (2015). Nanoscale co-based catalysts for low-temperature CO oxidation. Catal. Sci. Technol..

[10] Khan NA, Hasan Z, Jhung SH (2013). Adsorptive removal of hazardous materials using metal–organic frameworks (MOFs): a review. J. Hazard. Mater..

[11] Karra JR, Walton KS (2008). Effect of open metal sites on adsorption of polar and nonpolar molecules in metal–organic framework Cu-BTC. Langmuir.

[12] Khan NA, Jhung SH (2012). Remarkable adsorption capacity of CuCl_2_-loaded porous vanadium benzenedicarboxylate for benzothiophene. Angew. Chem. Int. Ed..

[13] Britt D, Tranchemontagne D, Yaghi OM (2008). Metal–organic frameworks with high capacity and selectivity for harmful gases. Proc. Natl. Acad. Sci. U.S.A..

[14] Irvine DJ, McCluskey JA, Robinson IM (2000). Fire hazards and some common polymers. Polym. Degrad. Stab..

[15] Dasari A, Yu Z-Z, Cai G, Mai Y-W (2013). Recent developments in the fire retardancy of polymeric materials. Prog. Polym. Sci..

[16] Morgan AB, Gilman JW (2013). An overview of flame retardancy of polymeric materials: application, technology, and future directions. Fire Mater..

[17] Carpentier F, Bourbigot S, Bras ML, Delobel R, Foulon M (2000). Charring of fire retarded ethylene vinyl acetate copolymer–magnesium hydroxide/zinc borate formulations. Polym. Degrad. Stab..

[18] Wang Z, Qu B, Fan W, Huang P (2001). Combustion characteristics of halogen-free flame-retarded polyethylene containing magnesium hydroxide and some synergists. J. Appl. Polym. Sci..

[19] Gao Y, Wu J, Wang Q, Wilkieb CA, O’Hare D (2014). Flame retardant polymer/layered double hydroxide nanocomposites. J. Mater. Chem. A.

[20] Liu J, He Y, Chang H, Guo Y, Li H, Pan B (2020). Simultaneously improving flame retardancy, water and acid resistance of ethylene vinyl acetate copolymer by introducing magnesium hydroxide/red phosphorus co-microcapsule and carbon nanotube. Polym. Degrad. Stab..

[21] Wang X, Kalalia EN, Wan J-T, Wang D-Y (2017). Carbon-family materials for flame retardant polymeric materials. Prog. Polym. Sci..

[22] Kim H, Kim DW, Vasagar V, Ha H, Nazarenko S, Ellison CJ (2018). Polydopamine-graphene oxide flame retardant nanocoatings applied via an aqueous liquid crystalline scaffold. Adv. Funct. Mater..

[23] Bao C, Song L, Wilkie CA, Yuan B, Guo Y, Hu Y, Gong X (2012). Graphite oxide, graphene, and metal-loaded graphene for fire safety applications of polystyrene. J. Mater. Chem..

[24] Xuan W, Zhu C, Liu Y, Cui Y (2012). Mesoporous metal–organic framework materials. Chem. Soc. Rev..

[25] Hou Y, Hu W, Gui Z, Hu Y (2017). A novel Co(II)-based metal–organic framework with phosphorus-containing structure: build for enhancing fire safety of epoxy. Compos. Sci. Technol..

[26] Howarth AJ, Liu Y, Li P, Li Z, Wang TC, Hupp JT, Farha OK (2016). Chemical, thermal and mechanical stabilities of metal–organic frameworks. Nat. Rev. Mater..

[27] Yuan S, Feng L, Wang K, Pang J, Bosch M (2018). Stable metal–organic frameworks: design, synthesis, and applications. Adv. Mater..

[28] Pan Y, Liu Y, Zeng G, Zhao L, Lai Z (2011). Rapid synthesis of zeolitic imidazolate framework-8 (ZIF-8) nanocrystals in an aqueous system. Chem. Commun..

[29] Yin H, Kim H, Choi J, Yip ACK (2015). Thermal stability of ZIF-8 under oxidative and inert environments: a practical perspective on using ZIF-8 as a catalyst support. Chem. Eng. J. (Amsterdam, Neth.).

[30] Qin J, Wang S, Wang X (2017). Visible-light reduction CO_2_ with dodecahedral zeolitic imidazolate framework ZIF-67 as an efficient co-catalyst. Appl. Catal. B.

[31] Valenzano L, Civalleri B, Chavan S, Bordiga S, Nilsen MH (2011). Disclosing the complex structure of UiO-66 metal organic framework: a synergic combination of experiment and theory. Chem. Mater..

[32] Garibay SJ, Cohen SM (2010). Isoreticular synthesis and modification of frameworks with the UiO-66 topology. Chem. Commun..

[33] Sai T, Ran S, Guo Z, Fang Z (2019). A Zr-based metal organic frameworks towards improving fire safety and thermal stability of polycarbonate. Compos. B.

[34] Kitao T, Zhang Y, Kitagawa S, Wang B, Uemura T (2017). Hybridization of MOFs and polymers. Chem. Soc. Rev..

[35] Tajima K, Aida T (2000). Controlled polymerizations with constrained geometries. Chem. Commun..

[36] Park I-H, Medishetty R, Lee H-H, Mulijanto CE, Quah HS, Lee SS, Vittal JJ (2015). Formation of a syndiotactic organic polymer inside a MOF by a [2 + 2] photo-polymerization reaction. Angew. Chem. Int. Ed..

[37] Gamage N-DH, McDonald KA, Matzger AJ (2016). MOF-5-polystyrene: direct production from monomer, improved hydrolytic stability, and unique guest adsorption. Angew. Chem. Int. Ed..

[38] Yang T, Shi GM, Chung T-S (2012). Symmetric and asymmetric zeolitic imidazolate frameworks (ZIFs)/polybenzimidazole (PBI) nanocomposite membranes for hydrogen purification at high temperatures. Adv. Energy Mater..

[39] Mahajan R, Koros WJ (2000). Factors controlling successful formation of mixed-matrix gas separation materials. Ind. Eng. Chem. Res..

[40] Elangovan D, Yuzay IE, Selke SEM, Auras R (2012). Poly(l-lactic acid) metal organic framework composites: optical, thermal and mechanical properties. Polym. Int..

[41] Hou Y, Hu W, Gui Z, Hu Y (2017). Preparation of metal–organic frameworks and their application as flame retardants for polystyrene. Ind. Eng. Chem. Res..

[42] Ji X, Xu Y, Zhang W, Cui L, Liu J (2016). Review of functionalization, structure and properties of graphene/polymer composite fibers. Compos. Part A Appl. Sci. Manuf..

[43] Wang X, Wang S, Wang W, Li H, Liu X (2020). The flammability and mechanical properties of poly (lactic acid) composites containing Ni-MOF nanosheets with polyhydroxy groups. Compos. B Eng..

[44] Zhang J, Li Z, Zhang L, Yang Y, Wang D-Y (2020). Green synthesis of biomass phytic acid-functionalized UiO-66-NH2 hierarchical hybrids toward fire safety of epoxy resin. ACS Sustain. Chem. Eng..

[45] Qi X-L, Zhou D-D, Zhang J, Hu S, Haranczyk M, Wang D-Y (2019). Simultaneous improvement of mechanical and fire-safety properties of polymer composites with phosphonate-loaded MOF additives. ACS Appl. Mater. Interfaces..

[46] Xia W, Mahmood A, Zou R, Xu Q (2015). Metal–organic frameworks and their derived nanostructures for electrochemical energy storage and conversion. Energy Environ. Sci..

[47] Lü Y, Wang Y, Li H, Lin Y, Jiang Z (2015). MOF-derived porous Co/C nanocomposites with excellent electromagnetic wave absorption properties. ACS Appl. Mater. Interfaces..

[48] Jiang Z, Li Z, Qin Z, Sun H, Jiao X, Chen D (2013). LDH nanocages synthesized with MOF templates and their high performance as supercapacitors. Nanoscale.

[49] Pan Y-T, Wan J, Zhao X, Li C, Wang D-Y (2017). Interfacial growth of MOF-derived layered double hydroxide nanosheets on graphene slab towards fabrication of multifunctional epoxy nanocomposites. Chem. Eng. J..

[50] Hou Y, Qiu S, Hu Y, Kundu CK, Gui Z, Hu W (2018). Construction of bimetallic ZIF-derived Co–Ni LDHs on the surfaces of GO or CNTs with a recyclable method: toward reduced toxicity of gaseous thermal decomposition products of unsaturated polyester resin. ACS Appl. Mater. Interfaces..

[51] Zhang Z, Qin J, Zhang W, Pan Y-T, Wang D-Y, Yang R (2020). Synthesis of a novel dual layered double hydroxide hybrid nanomaterial and its application in epoxy nanocomposites. Chem. Eng. J..

[52] Zhou X, Mu X, Cai W, Wang J, Chu F (2019). Design of hierarchical NiCo-LDH@PZS hollow dodecahedron architecture and application in high-performance epoxy resin with excellent fire safety. ACS Appl. Mater. Interfaces..

[53] Zhang Z, Li X, Yuan Y, Pan Y-T, Wang D-Y, Yang R (2019). Confined dispersion of zinc hydroxystannate nanoparticles into layered bimetallic hydroxide nanocapsules and its application in flame-retardant epoxy nanocomposites. ACS Appl. Mater. Interfaces..

[54] Zhou R, Ming Z, He J, Ding Y, Jiang J (2020). Effect of magnesium hydroxide and aluminum hydroxide on the thermal stability, latent heat and flammability properties of paraffin/HDPE phase change blends. Polymers.

[55] Pan Y-T, Zhang L, Zhao X, Wang D-Y (2017). Interfacial engineering of renewable metal organic framework derived honeycomb-like nanoporous aluminum hydroxide with tunable porosity. Chem. Sci..

[56] Shi X, Dai X, Cao Y, Li J, Huo C, Wang X (2017). Degradable poly(lactic acid)/metal–organic framework nanocomposites exhibiting good mechanical, flame retardant, and dielectric properties for the fabrication of disposable electronics. Ind. Eng. Chem. Res..

[57] Zheng Y, Lu Y, Zhou K (2019). A novel exploration of metal–organic frameworks in flame-retardant epoxy composites. J. Therm. Anal. Calorim..

[58] Hou Y, Xu Z, Yuan Y, Liu L, Ma S (2019). Nanosized bimetal–organic frameworks as robust coating for multi-functional flexible polyurethane foam: rapid oil-absorption and excellent fire safety. Compos. Sci. Technol..

[59] Chen W, Jiang Y, Qiu R, Xu W, Hou Y (2019). Investigation of UiO-66 as flame retardant and its application in improving fire safety of polystyrene. Macromol. Res..

[60] Li Y, Li X, Pan Y-T, Yu X, Song Y, Yang R (2020). Mitigation the release of toxic PH3 and the fire hazard of PA6/AHP composite by MOFs. J. Hazard. Mater..

[61] Xu B, Xu W, Wang G, Liu L, Xu J (2018). Zeolitic imidazolate frameworks-8 modified graphene as a green flame retardant for reducing the fire risk of epoxy resin. Polym. Adv. Technol..

[62] Xu W, Wang X, Wu Y, Li W, Chen C (2019). Functionalized graphene with Co-ZIF adsorbed borate ions as an effective flame retardant and smoke suppression agent for epoxy resin. J. Hazard. Mater..

[63] Zhou M, Cai L, Bajdich M, García-Melchor M, Li H (2015). Enhancing catalytic CO oxidation over Co_3_O_4_ nanowires by substituting Co^2+^ with Cu^2+^. ACS Catal..

[64] Yan N, Chen Q, Wang F, Wang Y, Zhong H, Hu L (2013). High catalytic activity for CO oxidation of Co_3_O_4_ nanoparticles in SiO_2_ nanocapsules. J. Mater. Chem. A.

[65] Zhang J, Kong Q, Yang L, Wang D-Y (2016). Few layered Co(OH)_2_ ultrathin nanosheets based polyurethane nanocomposites with reduced fire hazard: from eco-friendly flame retardance to sustainable recycling. Green Chem..

[66] Schartel B (2010). Phosphorus-based flame retardancy mechanisms-old hat or a starting point for future development?. Matererials.

[67] Xu B, Xu W, Liu Y, Chen R, Li W, Wu Y, Yang Z (2018). Surface modification of α-zirconium phosphate by zeolitic imidazolate frameworks-8 and its effect on improving the fire safety of polyurethane elastomer. Polym. Adv. Technol..

[68] Hou Y, Liu L, Qiu S, Zhou X, Gui Z, Hu Y (2018). DOPO-modified two-dimensional Co-based metal–organic framework: preparation and application for enhancing fire safety of poly(lactic acid). ACS Appl. Mater. Interfaces..

[69] Schartel B, Perret B, Dittrich B, Ciesielski M, Krämer J (2016). Flame retardancy of polymers: the role of specific reactions in the condensed phase. Macromol. Mater. Eng..

[70] Hou Y, Hu W, Zhou X, Gui Z, Hu Y (2017). Vertically aligned nickel 2-methylimidazole metal–organic framework fabricated from graphene oxides for enhancing fire safety of polystyrene. Ind. Eng. Chem. Res..

[71] Lin K-YA, Lee W-D (2016). Self-assembled magnetic graphene supported ZIF-67 as a recoverable and efficient adsorbent for benzotriazole. Chem. Eng. J..

[72] Kim D, Kim DW, Hong WG, Coskun A (2016). Graphene/ZIF-8 composites with tunable hierarchical porosity and electrical conductivity. J. Mater. Chem. A.

[73] Zhang M, Shi X, Dai X, Huo C, Xie J, Li X, Wang X (2018). Improving the crystallization and fire resistance of poly(lactic acid) with nano-ZIF-8@GO. J. Mater. Sci..

[74] Zhang J, Zhi L, Qi X-L, Wang D-Y (2020). Size tailored bimetallic metal–organic framework (MOF) on graphene oxide with sandwich-like structure as functional nano-hybrids for improving fire safety of epoxy. Compos. B Eng..

[75] Li Z, González AJ, Heeralala VB, Wang D-Y (2018). Covalent assembly of MCM-41 nanospheres on graphene oxide for improving fire retardancy and mechanical property of epoxy resin. Compos. B Eng..

[76] Hong N, Song L, Wang B, Stec AA, Hull TR, Zhan J, Hu Y (2014). Co-precipitation synthesis of reduced graphene oxide/NiAl-layered double hydroxide hybrid and its application in flame retarding poly(methyl methacrylate). Mater. Res. Bull..

[77] Li A, Xu W, Chen R, Liu Y, Li W (2018). Fabrication of zeolitic imidazolate frameworks on layered double hydroxide nanosheets to improve the fire safety of epoxy resin. Compos. Part A Appl. Sci. Manuf..

[78] Guo W, Nie S, Kalali EN, Wang X, Wang W (2019). Construction of SiO_2_@UiO-66 core–shell microarchitectures through covalent linkage as flame retardant and smoke suppressant for epoxy resins. Compos. B Eng..

[79] Bourbigot S, Duquesne S (2017). Fire retardant polymers: recent developments and opportunities. J. Mater. Chem..

[80] Bartholmai M, Schartel B (2004). Layered silicate polymer nanocomposites: new approach or illusion for fire retardancy? Investigations of the potentials and the tasks using a model system. Polym. Adv. Technol..

[81] Zhang J, Li Z, Zhang L, Molleja J, Wang D-Y (2019). Bimetallic metal–organic framework and graphene oxide nano-hybrids induced carbonaceous reinforcement towards fire retardant epoxy: a novel alternative carbonization mechanism. Carbon.

[82] Zhang L, Chen S, Pan Y-T, Zhang S, Nie S (2019). Nickel metal–organic framework derived hierarchically mesoporous nickel phosphate toward smoke suppression and mechanical enhancement of intumescent flame retardant wood fiber/poly(lactic acid) composites. ACS Sustain. Chem. Eng..

[83] Huang R, Guo X, Ma S, Xie J, Xu J, Ma J (2020). Novel phosphorus-nitrogen-containing ionic liquid modified metal–organic framework as an effective flame retardant for epoxy resin. Polymers.

